# Melanopsin Driven Light Responses Across a Large Fraction of Retinal Ganglion Cells in a Dystrophic Retina

**DOI:** 10.3389/fnins.2020.00320

**Published:** 2020-04-03

**Authors:** Cyril G. Eleftheriou, Phillip Wright, Annette E. Allen, Daniel Elijah, Franck P. Martial, Robert J. Lucas

**Affiliations:** ^1^Burke Neurological Institute at Weill Cornell Medicine, White Plains, NY, United States; ^3^Faculty of Life Sciences, University of Manchester, Manchester, United Kingdom

**Keywords:** melanopsin, intrinsically photosensitive retinal ganglion cell, retina, retinal degeneration, multi-electrode array, pharmacology, retinal ganglion cells

## Abstract

Intrinsically photosensitive retinal ganglion cells (ipRGCs) express the photopigment melanopsin and project to central targets, allowing them to contribute to both image-forming and non-image forming vision. Recent studies have highlighted chemical and electrical synapses between ipRGCs and neurons of the inner retina, suggesting a potential influence from the melanopsin-born signal to affect visual processing at an early stage of the visual pathway. We investigated melanopsin responses in ganglion cell layer (GCL) neurons of both intact and dystrophic mouse retinas using 256 channel multi-electrode array (MEA) recordings. A wide 200 μm inter-electrode spacing enabled a pan-retinal visualization of melanopsin’s influence upon GCL activity. Upon initial stimulation of dystrophic retinas with a long, bright light pulse, over 37% of units responded with an increase in firing (a far greater fraction than can be expected from the anatomically characterized number of ipRGCs). This relatively widespread response dissipated with repeated stimulation even at a quite long inter-stimulus interval (ISI; 120 s), to leave a smaller fraction of responsive units (<10%; more in tune with the predicted number of ipRGCs). Visually intact retinas appeared to lack such widespread melanopsin responses indicating that it is a feature of dystrophy. Taken together, our data reveal the potential for anomalously widespread melanopsin responses in advanced retinal degeneration. These could be used to probe the functional reorganization of retinal circuits in degeneration and should be taken into account when using retinally degenerate mice as a model of disease.

## Introduction

Soon after their discovery as a new class of photoreceptor in the retina (Berson et al., 2002; Hattar et al., 2002), melanopsin expressing intrinsically photosensitive retinal ganglion cells (ipRGCs) were suggested to have intra-retinal projections (Sekaran et al., 2003). It has since become clear that ipRGCs impact retinal activity through at least two pathways: gap junction signaling to wide field amacrine cells (ACs) (Muller et al., 2010; Reifler et al., 2015) and excitatory projections to dopaminergic ACs (Zhang et al., 2008). These circuits mean that, although ipRGCs represent a small fraction (<6%) of RGCs (Hughes et al., 2013), their intra-retinal influence can be substantial. Thus, each wide field AC contacts other neurons over several millimeters (Lin and Masland, 2006) and dopamine diffuses through to all retinal layers, reducing gap junction coupling in photoreceptor (Ribelayga et al., 2008), horizontal (Teranishi et al., 1983), bipolar (Kothmann et al., 2009), amacrine (Hampson et al., 1992), and retinal ganglion cells (Arroyo et al., 2016) (for review see Bloomfield and Volgyi, 2009). Given the capacity of at least some ipRGCs to act as irradiance detectors (Dacey et al., 2005; Wong et al., 2005; Schmidt and Kofuji, 2009; Davis et al., 2015; Storchi et al., 2015), their intra-retinal connectivity has been considered as a mechanism for adjusting retinal function according to ambient light levels. Accordingly, there is evidence of ipRGC influences over visual response characteristics in both mice and humans (Hankins and Lucas, 2002; Allen et al., 2014; Allen and Lucas, 2016; Milosavljevic et al., 2018; Allen et al., 2019).

Intrinsically photosensitive retinal ganglion cells survive outer retinal degeneration and retain their ability to respond to light and drive non-image-forming responses (Yoshimura and Ebihara, 1996). Mouse models of retinal dystrophy have been extensively used in the study of ipRGCs and retinal degeneration (Panda et al., 2003; Sekaran et al., 2003), as well as in the development of retinal rescue strategies (Bi et al., 2006; Mathieson et al., 2012). However, ipRGCs and their intra-retinal influence are not always considered in the etiology of retinal degeneration or in the analysis of efficacy for experimental therapies. ipRGCs can support light driven c-Fos induction in the inner retina (Semo et al., 2016) and spiking (Atkinson et al., 2013) activity in ACs. However, although retinal connectivity has been shown to undergo severe remodeling with disease progression (Marc et al., 2003; Jones et al., 2012) the implications for ipRGCs’ intra-retinal input remain unexplored. Here we report that melanopsin signaling appears in a large fraction of ganglion cell layer (GCL) neurons in the dystrophic retina. Comparison with visually intact retinas indicates that this is a feature of the dystrophic state. Our data imply that intra-retinal signaling of ipRGCs is altered by degeneration.

## Materials and Methods

### Animals

All experiments were in accordance with the United Kingdom Animals (Scientific Procedures) Act (1986). Recordings were performed on eight *Pde6b*^*rd*1/*rd*1^; *Cnga3*^–/–^ with a C57Bl6 background (aged P80–P120), four *Pde6b*^*rd*1/*rd*1^ (aged P77–P215), six C57Bl6 (wild type control, aged P80–P100), six C57Bl6 *Opn4*^–/–^ (non-degenerated melanopsin knockout, aged P80-P100), and three *Pde6b*^*rd*1/*rd*1^; *Opn4*^–/–^ mice (aged P100-P120). The *Pde6b*^*rd*1/*rd*1^; *Cnga3*^–/–^ expressed a rodless + coneless phenotype, with most rods dead by the end of the second post-natal month and cones being functionless from birth due to the knockout of the *Cnga3* gene (Chang et al., 2002). The use of this phenotype abolishes the potential for residual cone responses, which survive rod death until post-natal day 90 in the *Pde6b*^*rd*1/*rd*^1 (rd1) model (Carter-Dawson et al., 1978; Lin et al., 2009). The *Pde6b*^*rd*1/*rd*1^; *Opn4*^–/–^ mice carry the rd1 82 loss of function mutation in the gene encoding the phosphodiesterase β6 subunit (*Pde6b*) that abolishes rod photo-transduction and causes rod, and subsequent cone cell death, with both types of photoreceptors dead by postnatal week 10 (Hart et al., 2005), and knockout of the melanopsin gene (*Opn4*) rendering melanopsin driven light transduction impossible (Lucas et al., 2003). We repeated some experiments in the widely used *Pde6b*^*rd*1/*rd*1^ mouse model over a range of ages (P77–P215), to confirm our observations were not specific to the combination of the *Pde6b* mutation and *Cnga3* knockout. In all of the following experiments, one retina was used per animal and the data were averaged across all age ranges. We did not systematically determine whether the fraction of responding RGCs per retina changed with age but the appearance of anomalously widespread responses was observed in melanopsin sufficient dystrophic models aged 77 days and above.

### *Ex vivo* Electrophysiology

Retinas were isolated as described in previous studies (Davis et al., 2015; Procyk et al., 2015) and then mounted onto a 256-channel multi-electrode array (256MEA200/30iR-ITO, Multichannel Systems) with the GCL facing down onto the electrodes. Electrophysiological signals were filtered (200 Hz high pass) and recorded in the form of spikes at a sampling rate of 25 kHz using MC_Rack software (Multi Channel Systems) through a USB-MEA256 amplifier. The retinal explant was superfused with carboxygenated (95% CO_2_/5% CO_2_) aCSF (artificial cerebro-spinal fluid, with concentrations in mM: 118 NaCl, 25 NaHCO3, 1 NaH2PO4, 3 KCl, 1 MgCl2, 2 CaCl2, 10 C6H12O6, 0.5 L-glutamine) at a rate of 2.7 ml/min and maintained at 32°C. Recordings were performed after a 30 min waiting period for the neural activity to stabilize. Spikes were sorted in Offline Sorter (Plexon) and saved as timestamps exported to Matlab (The Mathworks) for analysis.

Full field light stimuli were delivered to the GCL from below by a 470 nm LED (PhlatLight, Luminus Devices) at a irradiance of 2 × 10^15^ melanopsin-effective photons/cm^2^/s. Stimulation epochs consisted of a 10 s light step followed by either 120 or 500 s of darkness (20 s light steps and 120 s of darkness for *Pde6b*^*rd*1/*rd*1^ animal cohort). Spatially modulated stimuli were delivered through an LCD projection system (HoloEye, Photonics AG) which lowered the irradiance to 1 × 10^14^ photons/cm^2^/s (Govardovskii et al., 2000), with a dark to light ratio of 1000. Such stimuli consisted of a centrally positioned light bar spanning the height of the array and 400 μm thick. Each presentation lasted for 10 s with a 500 s interval of darkness and increased by 400 μm in width with each presentation (Figure 3A).

### Response Analysis

Responses were observed in NeuroExplorer and then further analyzed in Matlab, using a set of custom written scripts. Melanopsin responses were classified as positive when the time-averaged firing rate (500 ms time bins) in the 20 s following stimulus onset was higher than the mean + 2SD (over six repeated stimulations) across the 20 s preceding the stimulus. Peri-stimulus time histograms (PSTHs) were generated in Matlab with 500 ms bins (10 ms bins when studying outer-retinal photoreceptor responses), imported as number matrices into Prism 6 (GraphPad Software Inc.) where they were edited into images. Onset and offset latencies were calculated by smoothing the averaged timestamps with a two-bin sliding window and detecting when threshold was crossed.

## Results

### Widespread Light Responses in Degenerated Retinas

**FIGURE 1:**
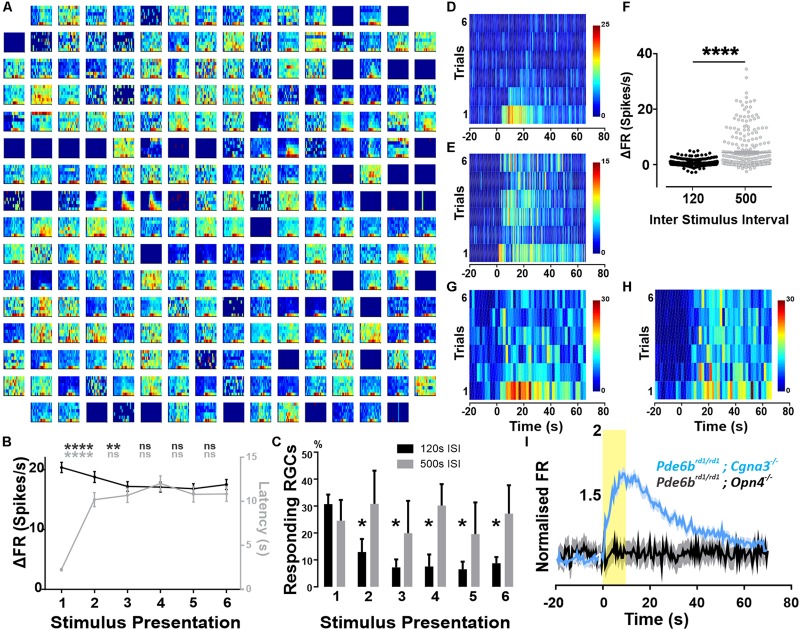
Widespread melanopsin driven responses in the degenerated retina. Recordings from *Pde6b*^*rd*1/*rd*1^; *Cgna3*^–/–^ retinas **(A–H)**. **(A)** Example trial bin count (TBC, 2 s bins, seven trials) responses to 10 s full field light pulses (melanopsin log 15 photons/cm^2^/s) intercalated with 120 s of darkness. Data shown are multi-unit activity (MUA) from a single retina. **(B)** Average ΔFR and onset latencies (±SEM) of neurons classified as responsive, over six stimulus presentations. Friedman test with *post hoc* Dunn’s multiple comparisons test between subsequent repeats. ****p < 0.0001, **p = 0.0013. *N* = 515 neurons. **(C)** Average (+SEM) percentage of responding neurons per retina on each stimulation presentation according to ISI. No significant difference between both groups using two-way RM ANOVA with *post hoc* Sidak’s multiple comparison test. Significant decrease in number of responses compared to first stimulation for 120 s ISI, *p = 0.0335 (stim2), *p = 0.0239 (stim3), *p = 0.0143 (stim4), *p = 0.0227 (stim5), *p = 0.289 (stim6). No significant reduction for 500 s ISI. *N* = 4 retinas. **(D)** Example TBC of responding neuron classified as decaying: displays an infra-threshold ΔFR from the third stimulus repeat onward. 120 s ISI. **(E)** Example TBC of neuron classified as robust: maintains supra-threshold ΔFR throughout all stimulus repeats. 120 s ISI. **(F)** Average ΔFR of all initially responsive neurons, on the sixth stimulus presentation for inter-stimulus intervals (ISI) of either 120 or 500 s. Wilcoxon matched-pairs signed rank test, *n* = 282 neurons from four retinas, ****p < 0.0001. **(G,H)** Example TBCs of a decay-classified neuron showing robust response characteristics with 500 s stimulus ISI **(H)** compared to 120 s ISI **(G)**. **(I)** PSTH (±SEM) of all cells presented with a 10 s light pulse (log 15 photons/cm^2^/s) for both *Pde6b*^*rd*1/*rd*1^; *Opn4^–/–^* (black, MUA activity) and *Pde6b*^*rd*1/*rd*1^; *Cgna3*^–/–^ (blue, single unit activity) phenotypes, normalized to average baseline firing rate. Yellow bar represents stimulus duration (10 s).

Extracellular recordings of GCL neurons from eight *Pde6b*^*rd*1/*rd*1^;*Cnga3*^–/–^ retinas displayed spontaneous spiking, with a mean firing rate of 6.80 Hz (±0.43 SEM) in the dark. When subjected to a 10 s full field light pulse (melanopsin log 15 photons/cm^2^/s), 37% of isolated single units (515/1384 units from eight retinas) displayed a sustained increase in firing (shown for a representative preparation in Figure 1A). This widespread response shared the established properties of melanopsin-driven activity, having a relatively long onset latency (1.2 s median) and persisting not only throughout light presentation but also for tens of seconds after stimulus termination (response duration range = 23–35 s).

The magnitude and extent of responses to the 10 s light pulse were strongly reduced by repeated presentation of the stimulus, even at the relatively long inter-stimulus interval (ISI) of 120 s. Thus, across all cells classified as responsive to the first presentation, increases in firing rate decayed over subsequent repeats (Figure 1B, *p* < 0.0001 for second stimulation, *p* = 0.0013 for third stimulation, *n* = 515 neurons Friedman test with Dunn’s multiple comparisons). For 75% of initially responding cells, this loss in responsiveness was sufficient to render them no-longer light responsive according to our objective criterion (see section “Materials and Methods”; Figures 1C,D, two-way RM ANOVA with *post hoc* Sidak’s multiple comparison test, *n* = 4 retinas). As a result, only 25% of light responsive units retained supra-threshold ΔFRs across repeated stimulus presentations (Figure 1E). Among this population of robustly responsive neurones, onset latency increased significantly for the second stimulus presentation but stabilized for subsequent presentations (Figure 1B, *p* < 0.0001, *n* = 515 neurons).

To explore the poor reproducibility of many responses, we increased the ISI to 500 s. This was sufficient to retain widespread responses across multiple presentations (Figure 1C), and enhanced response amplitude for later presentations (shown for representative unit in Figures 1G,H and for the last presentation to neurons classified as responsive on the first presentation Figure 1F, *p* < 0.0001, Wilcoxon matched-pairs signed rank test, *n* = 282 neurons).

As *Pde6b*^*rd*1/*rd*1^ mice are the most widely used animal model of retinal degeneration. We next set out to confirm that the widespread melanopsin responses observed in *Pde6b*^*rd*1/*rd*1^; *Cnga3*^–/–^ were replicated in the absence of the additional Cnga3 knockout. In a cohort of *Pde6b*^*rd*1/*rd*1^ animals, we observed an average of 41.2% (±9.39 SEM) responding neurons, with 31.4% (±8.70 SEM) showing repeated responses over multiple stimulus presentations and the remainder responding only to the first few light pulses (Supplementary Figure S1). These findings confirm that the widespread melanopsin light responses observed in *Pde6b*^*rd*1/*rd*1^; *Cnga3*^–/–^ are a feature also of the more commonly used *Pde6b*^*rd*1/*rd*1^, although they may be more robust to repeated presentation in the latter genotype.

To confirm that these light responses originate with melanopsin, we searched for them in retinally degenerate mice lacking melanopsin (*Pde6b*^*rd*1/*rd*1^; *Opn4*^–/–^ 250–300 post-natal days). In these melanopsin knockout animals, the same light-stimulation protocol produced significant changes in firing in only 19 out of 769 active channels (2.33 ± 0.54 responding channels per retina). Upon visual inspection, these were revealed as false-positives in which intrinsic oscillations in spontaneous firing produced changes in activity around the time of light exposure (not shown). Accordingly, while responses to a first 10 s stimulus were sufficiently widespread to appear in the combined firing rate of all units in *Pde6b*^*rd*1/*rd*1^; *Cnga3*^–/–^ GCLs this was not the case in retinas lacking melanopsin (Figure 1I).

To determine whether the widespread GCL melanopsin response revealed by our stimuli in dystrophic retinas was also apparent in the intact retina, we attempted to recreate this behavior in non-dystrophic retinas with and without pharmacological deafferentation of the inner retina.

**FIGURE 2:**
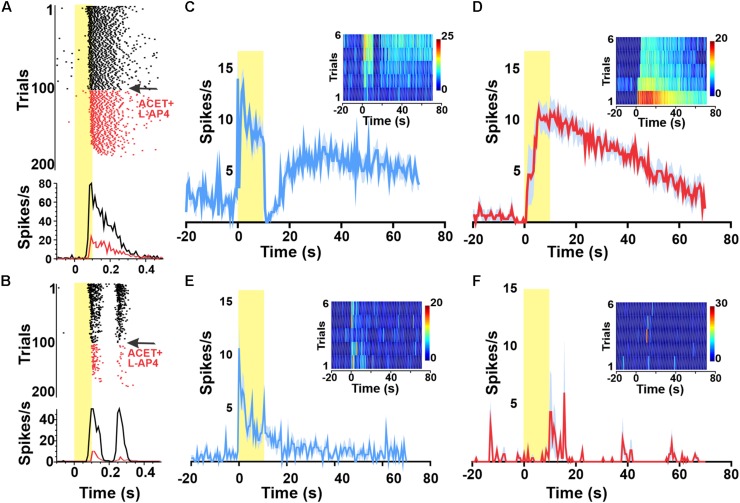
Widespread melanopsin responses are not observed in the intact retina. Raster plots and PSTHs of melanopsin-responsive unit **(A)** as well as a representative ON–OFF unit **(B)** exposed to a 1 Hz 100 ms flash (log 15 photons/cm^2^/s at time 0) reveal progressive loss of responses to this short duration stimulus following wash in of 100 μM DL-AP4 + 2 μM ACET starting at trial 100 (indicated in red). By trial 200, neither unit is responsive. Red PSTHs are of the last 30 stimulus presentations. **(C–F)** PSTHs and TBC (inserts) responses to 10/120 s stimuli of example a GCL unit (from **A**) classified as melanopsin-responsive from a wild type (wt) animal before **(C)** and after **(D)** pharmacological deafferentation from outer-retinal photoreceptors. PSTHs (±SEM) and TBC (inserts) responses to 10/120 s stimuli of example unit from a melanopsin knockout animal with a sustained response before **(E)** but not after **(F)** pharmacological deafferentation.

We confirmed the ability of a combination of the mGluR6 agonist DL-AP4 (100 μM, to block light responses in ON BCs) and KA-glutamatergic antagonist ACET (2 μM to block light responses in OFF BCs) to achieve this deafferentation by showing that they abolished responses to a 1 Hz flash stimulus (Figures 2A,B). We found that presenting the 10 s pulse stimulus with 120 s ISI (following 30 min of dark adaptation) indeed elicited melanopsin-like sustained responses from a subset of units before (Figure 2C) and during pharmacological deafferentation (Figure 2D). Under both conditions, the fraction of melanopsin-like responses for the first presentation was far lower than in dystrophic retinas (averages of 5.3% in aCSF and 1.9% under pharmacological deafferentation). Increasing the ISI to 500 s did not significantly change this outcome with only 3.2% of units showing a melanopsin like response in the wild type retina. As such, the high number of melanopsin-like responses appears to be a feature of the degenerated retina.

To confirm that our estimate of the number of melanopsin responsive units was not substantially impacted by misclassification of rod/cone signals, we repeated these experiments in *Opn4^–/–^* mice. We observed a small number of sustained responses in non-degenerated melanopsin knock-out retinas (Figure 2E), but these lacked the persistent firing after lights off expected for melanopsin-driven responses (Figure 2B) and were abolished by pharmacological deafferentation (Figure 2F).

### Melanopsin-Driven Responses Rely on Local Light Absorption

**FIGURE 3:**
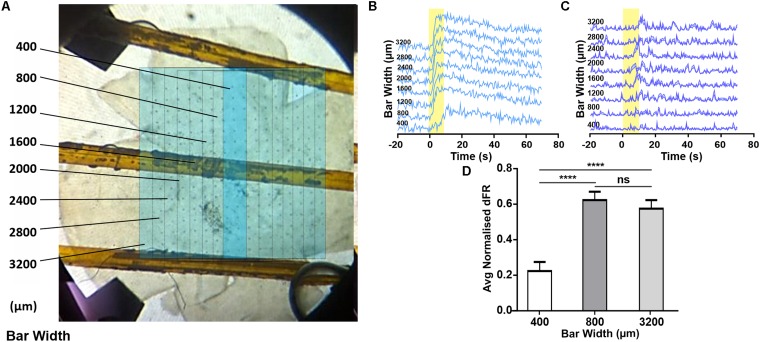
Changes in melanopsin-driven responses depending on size of illumination in the degenerated retina. **(A)** Photograph of retina on MEA, overlaid with a representation of the bar stimulus of increasing width (dark blue bar shows minimal width with sequential increases in width indicated with fine lines on light blue background; figures to left give width in μm). **(B,C)** Example PSTHs (normalized FR ± SEM) of cells requiring an 800 **(B)** and 1200 μm **(C)** wide stimulus to reach maximal response firing rate. **(D)** Mean (+SEM) change in firing rate as a function of the stimulus bar width. Friedman test with *post hoc* Dunn’s multiple comparisons test with *N* = 60 neurons; ****p < 0.0001.

The appearance of a widespread melanopsin response raises the question of whether this requires coordinated activity of multiple ipRGCs over large portions of the retina. To explore this possibility we attempted to restrict light exposure to a defined portion of the retina (note that for light pulse stimuli of the type used here, even moderate light scattering ensures that parts of the retina outside of that under direct stimuli also receive a light step albeit of much smaller magnitude). Using an LCD filter we presented a bar (10^14^ photons/cm^2^/s targeting melanopsin) 3.2 mm in height centered around the middle two columns of the MEA. We started with this bar at 400 μm (equivalent to the maximum size of an ipRGC’s dendritic field; Estevez et al., 2012) and sequentially increased it by 400 μm (Figure 3A). If each response of units recorded from the middle two electrode columns originated with the activity of a single ipRGC, we might expect their response amplitude to increase as bar width increased from 400–800 μm (to encompass more of the dendritic field of the local ipRGC), but to stabilize for further increases in width. We did find some units whose responses increased with very wide bars (e.g., Figure 3C), but most units showed no further increases beyond bar widths of 800 μm (Figures 3B,D, Friedman test with *post hoc* Dunn’s multiple comparisons, *n* = 60 neurons). These findings suggest that most melanopsin responses are driven by the activity of local ipRGCs rather than a wider syncytium.

### Different Synaptic Blockers Eliminate Light Responsiveness in Different Fractions of Neurons

**FIGURE 4:**
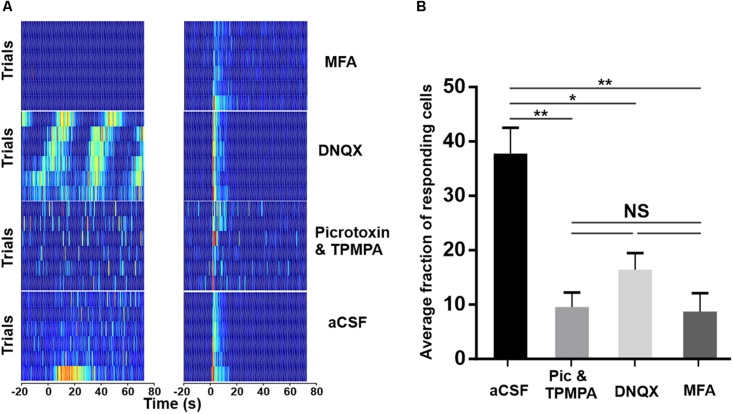
Pharmacological isolation of ipRGCs in the degenerated retina. **(A)** Trial bin counts for two example units that responded to a 10 s light pulse (at time 0; ISI = 120 s) under aCSF (bottom) but then either retained (right) or lost (left) responses under pharmacological block of ionotropic GABA (Picrotoxin and TPMPA), AMPA-type glutamate (DNQX), or gap-junctions (MFA), respectively. **(B)** Mean (+SEM) fraction of units showing a light response under aCSF and after pharmacological blockade. Two-tailed Mann–Whitney test of averages between aCSF and Picrotoxin and TPMPA (*N* = 5 retinas, **p = 0.0081), DNQX (*N* = 4 retinas, *p = 0.0162), and MFA (*N* = 4 retinas, **p = 0.0031).

In an attempt to elucidate the circuitry underlying exportation of the melanopsin signal within the GCL, we attempted to pharmacologically block each of the reported routes by which light-evoked signals can exit ipRGCs. DNQX (100 μM) was bath applied to block the AMPA-type glutamatergic synapse between ipRGCs and dopaminergic ACs; meclofenamic acid (MFA, 50 μM) to block the gap junctions between ipRGCs and displaced widefield GABAergic ACs; and a cocktail of Picrotoxin with TPMPA (20 and 50 μM, respectively) was used to block the GABAergic output of displaced widefield ACs. In each case, we found that while some light responses survived, a substantial fraction were lost (Figure 4). In addition to inhibiting light responses, these agents also altered spontaneous activity (Supplementary Figure S2). Average spontaneous firing rate was decreased from 6.80 Hz ± 0.43 SEM under aCSF to 4.16 Hz ± 0.18 SEM under MFA and 5.60 Hz ± 0.37 SEM under DNQX application. Conversely, Picrotoxin and TPMPA increased the spontaneous firing rate to 11.15 Hz ± 0.38 SEM. In addition, inhibition of AMPA-type glutamatergic block by DNQX induced infra-slow oscillations (0.01–0.05 Hz, Supplementary Figure S3) previously characterized *in vivo* (Orlowska-Feuer et al., 2016) in a subset of units. Thus, while these data might imply that all known routes of signal transmission from ipRGCs to the surrounding retina contribute to the widespread melanopsin-driven light responses in the degenerate retina, we cannot exclude the alternative explanation that the impact of some drugs on light responses is secondary to their effect on other aspects of retinal circuitry in the dystrophic condition.

## Discussion

### Light Responses Recorded on the MEA Are Mostly From RGCs

We report here a large fraction of cells showing light responses, as increases in spike rate, in the GCL of degenerate retinas. Although RGCs are often perceived as the only spiking cells of the retina, there is a large body of literature demonstrating the capabilities of ACs to fire action potentials (Bloomfield, 1996; Tamalu and Watanabe, 2007), including displaced ACs of the GCL (Greschner et al., 2014). Since our recordings are of spiking cells in the retinal GCL, there is a risk that we may be reporting the firing of action potentials from these cells. High density MEAs allow the electrical visualization of axonal action potential propagation (Greschner et al., 2014) enabling positive identification of RGCs but our system does not allow us to make this distinction.

Nevertheless, we believe that at least a substantial majority of the melanopsin responses we record in the dystrophic retina are from retinal ganglion cells. Anatomical characterizations of AC populations estimate that displaced ACs constitute 60% of the neurons in the GCL (Jeon et al., 1998). A large proportion (66%) of these cells are Starburst ON ACs (Perez De Sevilla Muller et al., 2007) which only spike during development (Zhou and Fain, 1996). Spiking ACs observed in the adult GCL include the A17 and the Polyaxonal AC which account for, respectively, 3 and 7.5% of displaced ACs (Perez De Sevilla Muller et al., 2007), making these account for approximately 6.3% of GCL neurons. When randomly patching 3900 cells of the GCL, Reifler et al. (2015) found only 154 spiking ACs (3.9%) to be melanopsin driven. Based on these data, we can conservatively estimate that at least 90% of the spiking neurons we recorded to be RGCs.

### ipRGCs Drive Light-Responses in Non-Photosensitive RGCs

Our main finding, that melanopsin drives light responses in over 30% of RGCs in the degenerated retina was very surprising considering that melanopsin expressing ipRGCs represent <6% of RGCs (Hughes et al., 2013), and that the most light-responsive, M1, type are often present in the inner nuclear layer (Schmidt and Kofuji, 2009) and will thus not be sampled by our MEA recordings. This implies that the great majority of the GCL light responses reported here reflect intra-retinal signaling from ipRGCs in the INL and/or GCL. We might consider why such widespread light responses have not previously been reported given that there are numerous studies recording from the *Pde6b*^*rd*1/*rd*1^ retina. We found that the high proportion of responses was dependent on the use of a bright (log 15 photons/cm^2^/s) and long duration stimulus presented at long ISI, and was disrupted by inclusion of numerous pharmacological agents. Previous MEA recordings performed on explanted dystrophic retinae made use of lower irradiance stimuli (Jones et al., 2013) or combined their recordings with pharmacological blockers designed to reduce spontaneous activity (Tu et al., 2005; Zhu et al., 2007), thus inadvertently blocking ipRGC output pathways, which may explain why the unusually high proportion of melanopsin responses was not detected in those experiments.

One possible explanation for the widespread appearance of melanopsin responses is that more ganglion cells express melanopsin following degeneration, but this does not appear to be the case (Hughes et al., 2013; Semo et al., 2016). It follows that melanopsin signals must be exported within the retina to non-melanopsin expressing cells in the GCL. At least the vast majority of the melanopsin-driven responses that decay over multiple stimulus presentations likely therefore arise from one or more of the established routes by which ipRGC signals are exported to the neighboring retina. Here we have not been able to identify the routes via which the melanopsin signal is transmitted, but it would be interesting in future to do so and to establish the adaptation mechanisms by which it is inhibited following repeated exposure.

### Adaptive Nature of Light Responses

Gap junction coupling is plastic, operating on short, intermediate, or long-term timescales (O’Brien and Bloomfield, 2018). With short-term plasticity operating over milliseconds to seconds and long-term plasticity over minutes to hours. The decay we observed in the light response of many RGCs may be due to intermediate term plasticity. This corresponds to post-translational modifications, such as connexin phosphorylation/de-phosphorylation induced by intracellular cascades and initiated by neuromodulators like dopamine (Hampson et al., 1992; Mills and Massey, 1995), adenosine (Li et al., 2013), or nitric oxide (Jacoby et al., 2018), implemented over seconds to minutes and lasting from minutes to hours. As dopamine is a notorious mediator of light-adaptation via gap-junction de-phosphorylation (Bloomfield and Volgyi, 2009), we were expecting to abolish the decay in responses by blocking the retrograde connection to DACs. This was not the case, as we saw instead a decrease in light-responsive neurons. This may be because DACs of the dystrophic retina are anatomically (Ivanova et al., 2016b) and functionally (Atkinson et al., 2013) compromised, and thus unable to regulate gap junction phosphorylation. This results in heavy coupling between RGCs (Ivanova et al., 2016a) and elevated phosphorylation of connexin 36 (Ivanova et al., 2015), allowing the propagation of pathological oscillations from AII ACs into inner retinal circuits (Trenholm et al., 2012). It is possible that we observed in fact multiple different adaptation mechanisms, considering that nitric oxide and adenosine release is also mediated by ACs and that light activation to the first stimulus was likely propagated to many different neurons.

## Conclusion

In this study, we identify the potential for melanopsin to initiate spiking light responses in a high number of non-photosensitive neurons of the dystrophic retina. This should be taken into consideration when studying visual rescue strategies using dystrophic mouse models. We further identify a light-adaptation mechanism which reduces the extent of melanopsin responses and appears to be reversible by dark-adaptation. This highlights the ability of melanopsin to exert a dynamic influence over retinal circuitry in advanced stages of degeneration. The functional significance (if any) of this phenomenon remains to be determined, but it could plausibly provide some light-mediated neuro-protection or be a consideration in the nature of visual responses provided by therapeutic approaches to restoring vision in retinal degeneration.

## Data Availability Statement

The datasets generated for this study are available on request to the corresponding author.

## Ethics Statement

The animal study was reviewed and approved by the University of Manchester Animal Welfare Ethical Review Board and conducted under United Kingdom Home Office Animals Scientific Procedures e-Licensing (ASPeL).

## Author Contributions

CE and RL designed the study. CE and PW performed the experiments. CE, AA, and DE analyzed the data. CE and RL wrote the manuscript with input from all authors. CE and FM assembled the equipment.

## Conflict of Interest

The authors declare that the research was conducted in the absence of any commercial or financial relationships that could be construed as a potential conflict of interest.
