# Supplementary material for: Melanopsin Driven Light Responses Across a Large Fraction of Retinal Ganglion Cells in a Dystrophic Retina
Source: Front Neurosci. 2020 Apr 3;14:320. doi: 10.3389/fnins.2020.00320 (PMC7147324; doi:10.3389/fnins.2020.00320)
Supplement: Supplementary file 1 [file Table_1.pdf]

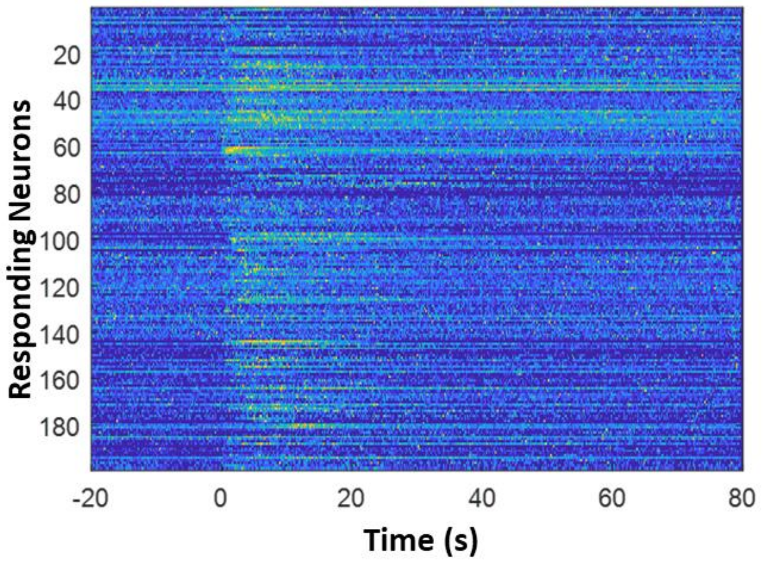

Figure S1: Population light-responses in *Pde6b*$^{rd1/rd1}$ retinas. Trial Bin Count plot of all neurons classified as light responsive in four *Pde6b*$^{rd1/rd1}$ retinas.

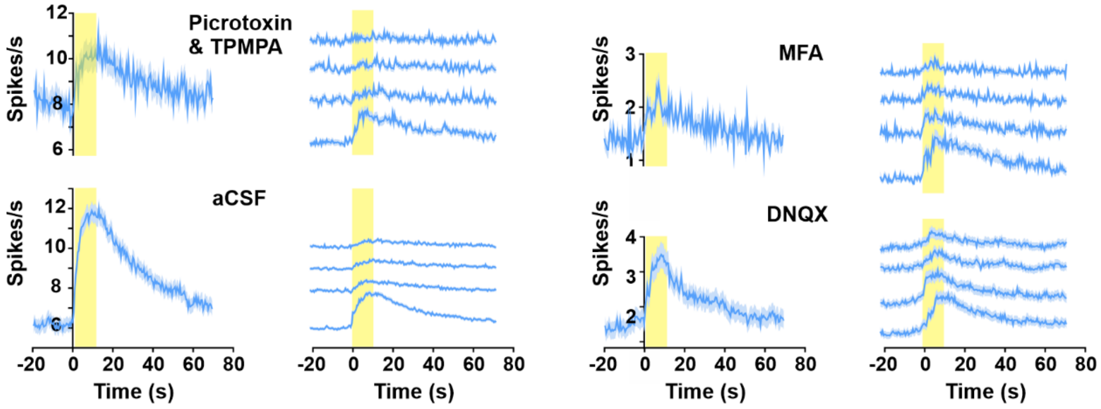

Figure S2: Population light-responses in *Pde6b*$^{rd1/rd1}$; *Cnga3*$^{-/-}$ retinas.under pharmacological blockades. Population PSTHs of all cells classified as light responsive (to a stimulus under no pharmacological influence) under all pharmacological conditions. Population PSTHs of first four presentations of a 10s pulse (at time 0) for all responding cells under each pharmacological condition shown to right, and individual population traces for the 4 presentations (first light pulse at bottom) shown to left.

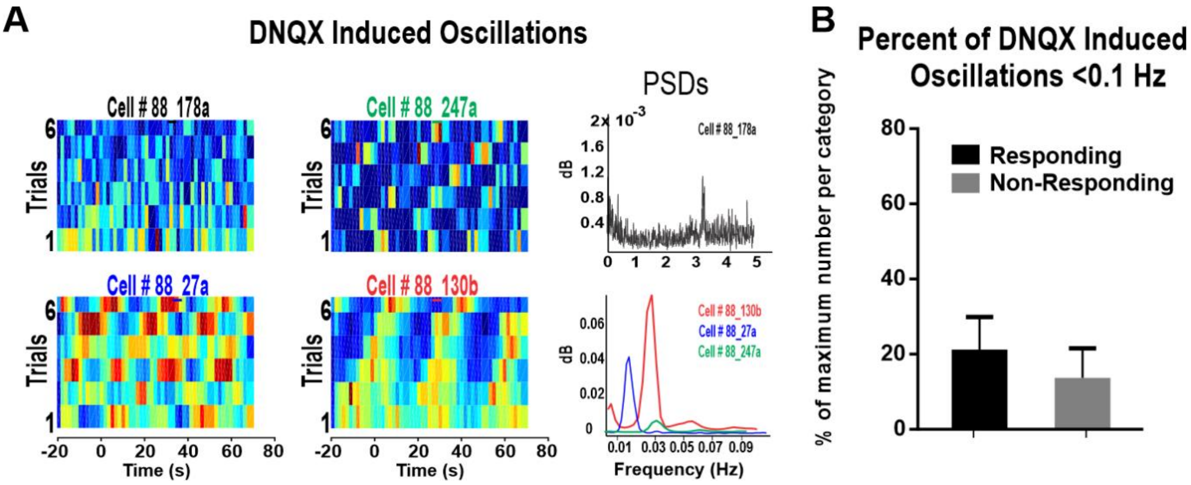

Figure S3: Potent and very slow oscillations in *Pde6b*$^{rd1/rd1}$*; Cnga3*$^{-/-}$ retina under DNQX influence. **A)** Example TBCs (Left) with associated PSDs (Right) of cells oscillating under the influence of 100 µM DNQX. **B)** Average proportion (±SEM) of cells responding (black bar) vs non-responding (grey bar) to light exhibiting potent and slow oscillation under DNQX influence.
